# A Case of Thyroid Carcinoma Showing Thymus-Like Differentiation With Breast Cancer Susceptibility Gene 2 Mutation: A Case Report and Literature Review

**DOI:** 10.7759/cureus.30655

**Published:** 2022-10-25

**Authors:** Masashi Kuroki, Hirofumi Shibata, Ryota Iinuma, Hiroshi Okuda, Toshimitsu Ohashi, Takenori Ogawa, Yukio Horikawa

**Affiliations:** 1 Otolaryngology - Head and Neck Surgery, Gifu University Graduate School of Medicine, Gifu, JPN; 2 Clinical Genetics Center, Gifu University Hospital, Gifu, JPN

**Keywords:** arid1a, brca2, cancer genome panel, thyroid cancer, intrathyroidal thymic carcinoma, castle

## Abstract

Carcinoma showing thymus-like differentiation (CASTLE) is a rare malignant tumor that originates from ectopic thymic or residual embryonic tissues. CASTLE is specified as a synonym for intrathyroidal thymic carcinoma.

The patient is a 66-year-old male. Surgery was performed on the thyroid tumor with tracheal infiltration, and pathological examination revealed CASTLE. Multidisciplinary treatment, including chemoradiotherapy, was performed for recurrent tumors, and he has been alive for 90 months since the initial treatment. The cancer genome panel identified mutations in AT-rich interaction domain 1A(*ARID1A*)and breast cancer susceptibility gene 2 (*BRCA2*), but there were no available clinical trials or recommended drugs. *BRCA2* may be involved in CASTLE. Herein, we review the literature and report the treatment method and gene mutation for recurrent metastatic cases of CASTLE, for which standard treatment has not been established.

## Introduction

Carcinoma showing thymus-like differentiation (CASTLE) is a rare malignant tumor with thymic epithelial differentiation, often originating from the thyroid gland. The fourth edition of the World Health Organization (WHO) classification specifies that it is a synonym for intrathyroidal thymic carcinoma in thyroid tumors [1]. We encountered a case of CASTLE originating in the thyroid gland and performed a cancer genome panel.

## Case presentation

A 66-year-old male visited the internal medicine department with hoarseness and a cough. Ultrasound revealed a hypoechoic tumor with extraglandular invasion in the left lobe of the thyroid, and the patient was referred to our hospital. The patient had a history of diabetes, dyslipidemia, hypertension, and hyperuricemia and had no family history of cancer.

**Figure 1:**
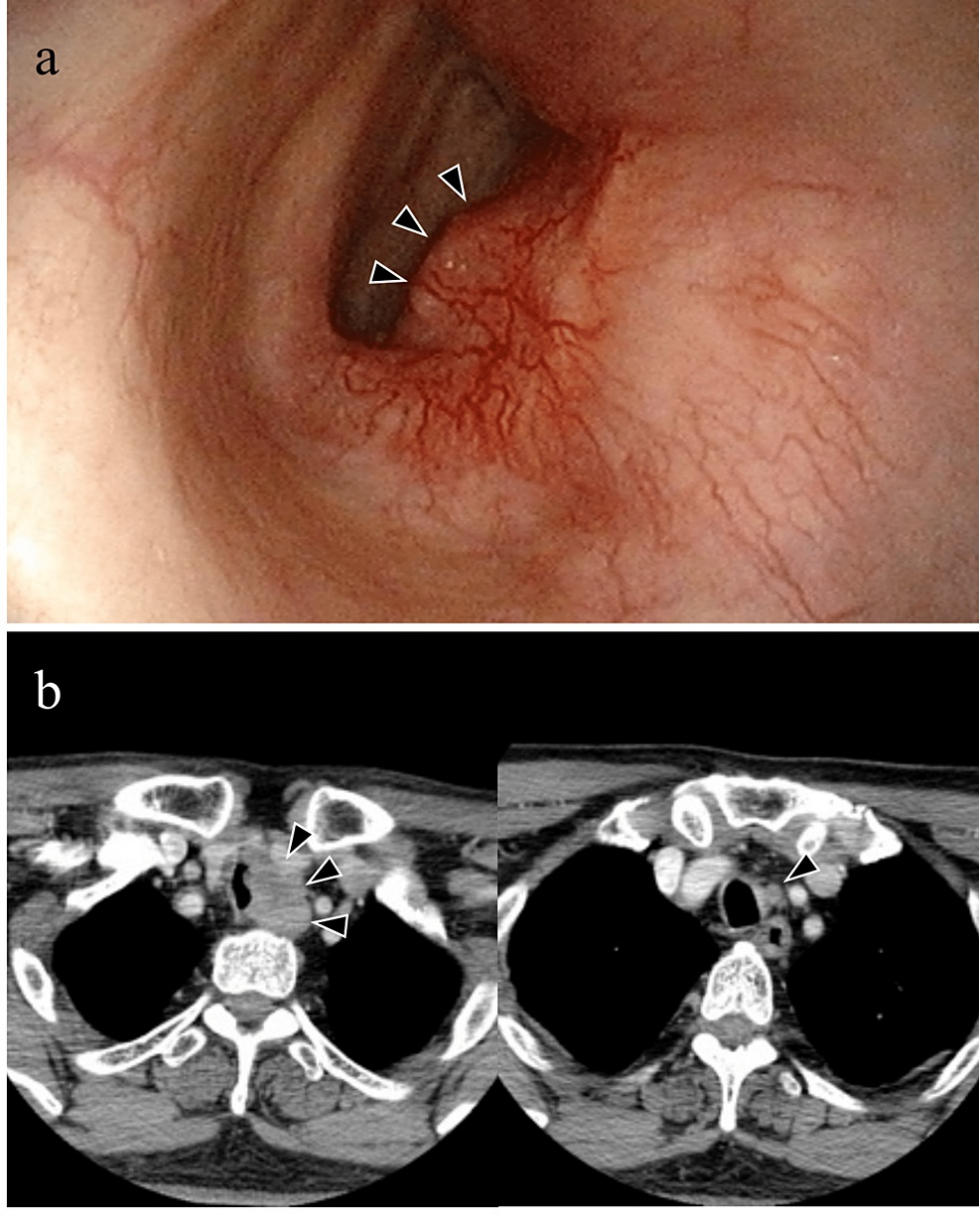
Preoperative and intraoperative findings a: Laryngeal endoscopy reveals paralysis of the left vocal cord, and arrowheads indicate a tumor protruding into the lumen of the left trachea. b: Computed tomography shows a 21 × 21 mm-sized low-concentration mass with an unclear boundary in the lower pole of the left lobe of the thyroid gland, and infiltration into the trachea was suspected. Arrowheads indicate the tumor region. Swelling of the left paratracheal lymph node was also observed.

Laryngeal endoscopy revealed paralysis of the left vocal cord and confirmed that the tumor protruded into the lumen of the left trachea (Figure 1a). Blood tests revealed normal levels of thyroid hormones, thyroglobulin, and various markers. Computed tomography revealed a low-density, ill-defined tumor measuring 21 × 21 mm in the lower pole of the left lobe of the thyroid gland. Tumor infiltration of the trachea was suspected. Swelling of the left paratracheal lymph node was also observed (Figure 1b). Fine needle aspiration cytology revealed atypical cells with a large nuclear/cytoplasmic (N/C) ratio and increased nuclear chromatin levels but almost no papillary agglomeration or follicular formation. An open biopsy was performed because it was difficult to distinguish. Based on the results of the biopsy, poorly differentiated thyroid cancer, neuroendocrine tumor, and thymus-like tumor were suspected. The tumor was staged as cT4aN1aM0, according to the classification of thyroid cancer. Total thyroidectomy, bilateral D1 dissection, cervical tracheal resection, and tracheal reconstruction were performed.

**Figure 2:**
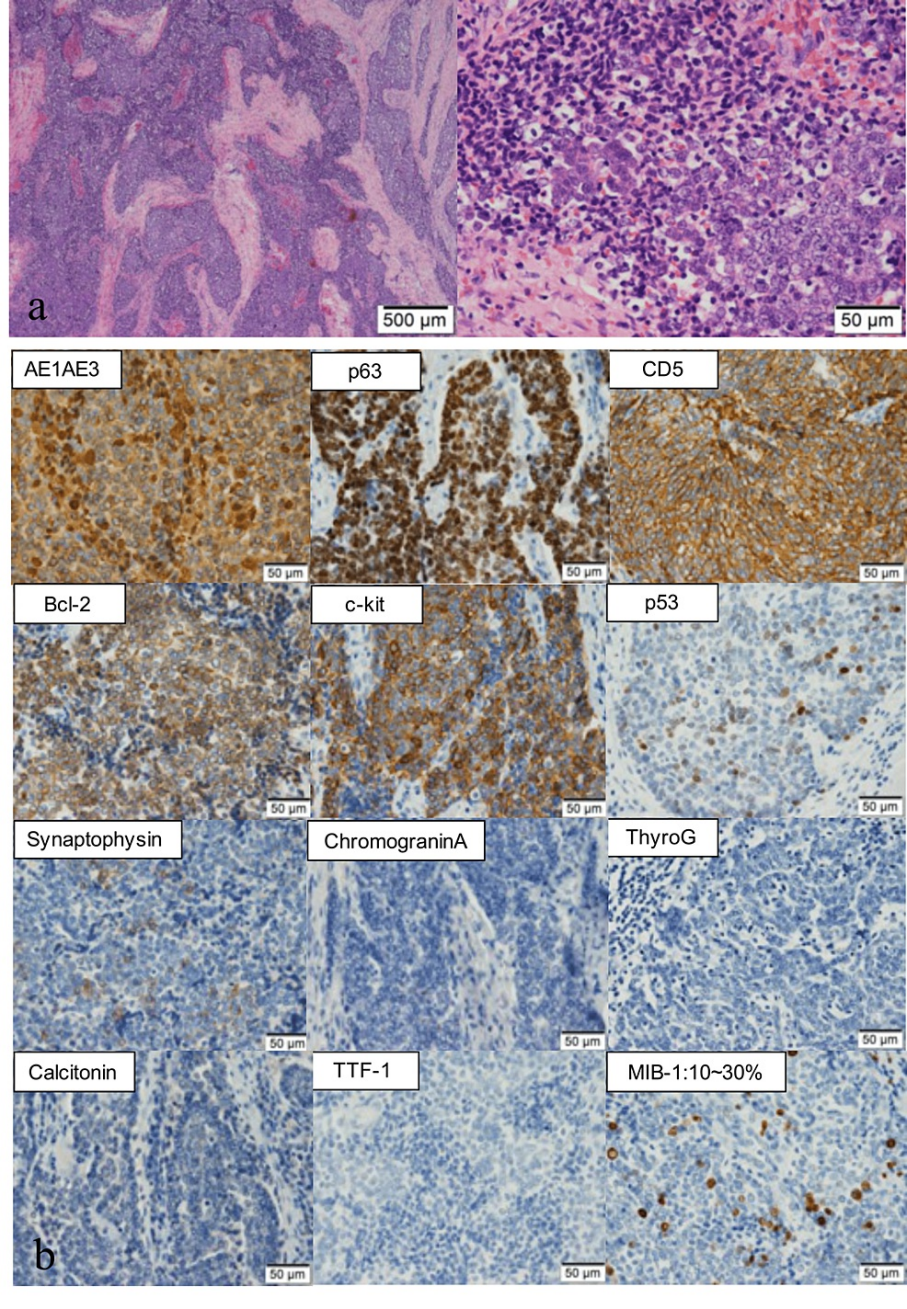
Postoperative histopathological findings a: Postoperative histopathological examination revealed that tumor cells with a high N/C ratio had infiltrated and proliferated in the form of alveolar lesions. b: Immunohistochemical staining shows cytokeratin (CK) AE1/AE3 (+), p63 (+), cluster of differentiation 5 (CD5) (+), S100 protein (S100P) (-), thyroid transcription factor 1 (TTF-1) (-), thyroglobulin (-), calcitonin (-), parathyroid hormone (PTH) (-), synaptophysin partially weak (+), chromogranin A (-), and CD56 (-), and the MIB-1 index was 10%-30%.

Postoperative histopathological examination revealed that tumor cells with a high N/C ratio had infiltrated and proliferated in the form of alveolar lesions (Figure 2a). Hyaline and lymphocyte-rich stroma infiltrated around the tumor follicle. Immunohistochemical staining showed cytokeratin (CK) AE1/AE3 (+), p63 (+), cluster of differentiation 5 (CD5) (+), S100 protein (S100P) (-), thyroid transcription factor 1 (TTF-1) (-), thyroglobulin (-), calcitonin (-), parathyroid hormone (PTH) (-), synaptophysin partially weak (+), chromogranin A (-), and CD56 (-), and MIB-1 index was 10%-30% (Figure 2b). Based on these results, the tumor was diagnosed as CASTLE (pT4a, pEx2, and pN1b).

Thirty-five months after the first operation, recurrence was detected in the left neck and mediastinal lymph nodes. Left neck and mediastinal dissections were performed. Re-recurrence occurred 45 and 62 months after the first surgery, and surgical resections were performed. However, re-recurrence occurred 77 months after the first surgery. Next-generation sequencing with the OncoGuide^TM^ NCC Oncopanel System (National Cancer Center, Tokyo, Japan, and Sysmex Corporation, Kobe, Japan) was performed to investigate tumor-specific mutations and identify appropriate chemotherapeutic drugs.

Sequencing was performed using formalin-fixed paraffin-embedded tissue from the metastatic lymph nodes, with patient consent. A total of 114 cancer-related mutations were detected using the OncoGuide^TM^ NCC Oncopanel System. The biomarker findings were stable microsatellite status and 0.8 mutations/megabase for tumor mutational burden. Gene mutations in AT-rich interaction domain 1A (*ARID1A*) and breast cancer susceptibility gene 2 (*BRCA2*) were found, and the variant allele frequencies were 32% and 40%, respectively. The ATR inhibitors BAY1895344 and M5520 (CX970) have been suggested to be effective against *ARID1A* mutations. However, there were no available clinical trials or recommended drugs (Table 1).

**Table 1:** Cancer genome panel findings The biomarker findings were stable microsatellite status and 0.8 mutations/megabase for the tumor mutational burden. Mutations in AT-rich interaction domain 1A (*ARID1A*) and breast cancer susceptibility gene 2 (*BRCA2*) were found, with allele frequencies of 32% and 40%, respectively.

Biomarker findings				
Microsatellite status			Stable	
Tumor mutational burden			0.8 mutations/megabase	
Genomic findings				
Gene	Alteration	Variant allele frequency (%)		Therapies with clinical benefit
ARID1A	A347fs*53	32		None
BRCA2	V2109I	40		None

Subsequently, concurrent chemoradiotherapy with cisplatin was performed, and the patient was alive with cancer 90 months after the first surgery.

## Discussion

CASTLE is defined as a malignant epithelial tumor of the thyroid gland with thyroid epithelial differentiation and an ectopic thyroid tumor in the thyroid gland [1]. CASTLE is a synonym for intrathyroidal thymic carcinoma, and other names are mentioned, including intrathyroidal epithelial thymoma, carcinoma showing thymus-like differentiation, primary thyroid thymoma, carcinoma showing thymus-like features, lymphoepithelioma, and CD5-positive thyroid carcinoma [2-7]. There have been some case reports of CASTLE in Japan and China. However, this frequency is rare, with 0.083% of thyroid tumors in Japan and 0.15% of thyroid tumors in China [7,8]. Most of the primary sites are the thyroid glands, but some papers have reported parotid gland-oriented cases [9-11]. The subjective symptoms were a neck mass and hoarseness due to recurrent laryngeal nerve palsy, and blood tests showed normal thyroid hormone levels [12].

The histopathological findings were similar to those of mediastinal thymic carcinoma accompanied by squamous epithelial characteristics. The histological grade is lower, and nuclear atypia is milder than that of squamous cell carcinoma [1]. Immunohistochemical staining was positive for CD5, c-kit, p63, BCL2, and calretinin and negative for TTF-1 and thyroglobulin, which are positive for differentiated thyroid cancers [13]. In addition, as in this case, CASTLE may have neuroendocrine properties, such as positive synaptophysin and chromogranin A [7,14]. CASTLE is histologically classified as squamous cell carcinoma type, lymphoepithelioma or basaloid type, and neuroendocrine carcinoma type [7]. In thymic cancer, squamous cell carcinoma type and basaloid carcinoma type have a better prognosis than lymphoepithelial-like carcinoma and neuroendocrine carcinoma type [15]. Based on partial synaptophysin-positive data, this case was correlated with the neuroendocrine tumor type.

Although the standard treatment for CASTLE has not been established, surgical treatments are performed in most resectable cases. Many reports have indicated the effectiveness of postoperative radiotherapy in locally advanced cases [12,16]. Chemotherapy is often selected according to the treatment of thymic carcinoma, but Lorenz et al. [10] reported a case of partial response to pembrolizumab. The prognosis after curative resection is relatively good, and the disease-specific survival rate is 90% at five years and 82% at 10 years [12].

There are only five reports of genetic testing for CASTLE (Table 2). Wang et al. [17] and Rajeshwari et al. [18] identified mutations in the epidermal growth factor receptor (*EGFR*), and Veits et al. [19] identified mutations in *EGFR* and platelet-derived growth factor receptor (*PDGFR*). Ishikawa et al. [9] performed whole-exome sequencing and identified somatic mutations in Fraser extracellular matrix complex subunit 1 (FRAS1)-related extracellular matrix 2 (*FREM2*), CDC-like kinase 3 (*CLK3*), discs large (DLG)-associated protein 1 (*DLGAP1*), nicotinamide adenine dinucleotide phosphate (NADPH) oxidase 1 (*NOX1*), and pregnancy-specific beta-1-glycoprotein 9 (*PSG9*). Wong et al. [11] performed an Oncomine^TM^ Comprehensive Cancer Panel (143 genes) and detected germline mutations in peroxisome proliferator-activated receptor γ (*PPARG*), *BRCA2*, and Notch receptor 1 (*NOTCH1*). *BRCA2* mutation was detected, as in our case, but neither of the cases reported by Wong et al. [11] nor our case had a family history of cancer. Principe et al. [20] reported a case in which tumor control was possible using the PARP inhibitor olaparib for metastatic thymoma with *BRCA2* mutation. Treatment targeting *BRCA2* may also be effective for CASTLE with *BRCA2* mutations. However, the frequency of the occurrence of CASTLE is extremely low, and it is hoped that cases involving genetic testing will be more common in the future.

**Table 2:** Report on carcinoma showing thymus-like differentiation (CASTLE) gene mutation Wang et al. [17] and Rajeshwari et al. [18] identified mutations in epidermal growth factor receptor (*EGFR*), and Veits et al. [19] identified mutations in *EGFR* and platelet-derived growth factor receptor (*PDGFR*). Ishikawa et al. [9] performed whole-exome sequencing and detected somatic mutations in Fraser extracellular matrix complex subunit 1 (FRAS1)-related extracellular matrix 2 (*FREM2*), CDC-like kinase 3 (*CLK3*), discs large (DLG)-associated protein 1 (*DLGAP1*), nicotinamide adenine dinucleotide phosphate (NADPH) oxidase 1 (*NOX1*), and pregnancy-specific beta-1-glycoprotein 9 (*PSG9*). Wong et al. [11] performed oncogene panel tests and detected germline mutations in peroxisome proliferator-activated receptor γ (*PPARG*), *BRCA2*, and Notch receptor 1 (*NOTCH1*). *BRCA2*: breast cancer susceptibility gene 2, *ARID1A*: AT-rich interaction domain 1A

Author	Year	Primary	Gene	Nucleotide	Protein
Veits et al. [19]	2014	Thyroid	EGFR	c.2361G>A	p.Q787Q
			PDGFR	c.1701A>G	p.P567P
Wang et al. [17]	2015	Thyroid	EGFR	c.2363G>A	p.Q787Q
Wong et al. [11]	2018	Parotid	PPARG	-	p.P12A
			BRCA2	-	p.I2490T
			NOTCH1	-	p.T2455A
Rajeshwari et al. [18]	2018	Thyroid	EGFR	-	p.T790M
Ishikawa et al. [9]	2021	Parotid	FREM2	c.2581G>T	p.V861F
			CLK3	c.1128C>A	p.F376L
			DLGAP1	c.882G>T	p.K294N
			NOX1	c.493G>A	p.V165M
			PSG9	c.430+4A>T	-
Our case		Thyroid	ARID1A	c.1037_10381nsG	p.A347fs*53
			BRCA2	c.6325G>A	p.V2109I

## Conclusions

In this report, we described a case of CASTLE originating from the thyroid gland and detailed a cancer gene panel for recurrent lesions. Gene mutations in *ARID1A* and *BRCA2* were identified; however, there were no available clinical trials or recommended drugs. This is the second report of CASTLE with a *BRCA2* mutation. For this disease, for which standard treatment has not been established, the accumulation of cases including genetic testing is awaited in the future.
